# Case Report: Termination of unplanned pregnancy led to rapid deterioration of non-small-cell lung cancer during osimertinib treatment

**DOI:** 10.3389/fonc.2023.1073938

**Published:** 2023-10-17

**Authors:** Qizhi Ma, Pei Shu, Kexun Zhou, Yongsheng Wang

**Affiliations:** ^1^ Division of Thoracic Tumor Multimodality Treatment, Cancer Center, State Key Laboratory of Biotherapy, West China Hospital, Sichuan University, Chengdu, China; ^2^ Department of Medical Oncology, Cancer Center, West China Hospital, Sichuan University, Chengdu, China

**Keywords:** pregnancy, non-small-cell lung cancer, epidermal growth factor receptor, tyrosine kinase inhibitor, deterioration

## Abstract

We present a case of a woman with non-small-cell lung cancer (NSCLC) who experienced disease progression during treatment with the epidermal growth factor receptor (EGFR)-tyrosine kinase inhibitor (TKI) osimertinib due to an unplanned pregnancy. Given the risk of tumor progression, the patient underwent an artificial abortion. However, disease deterioration occurred shortly after termination of the pregnancy, with severe chest pain, increased dyspnea, and pleural effusion. After positive rescue measures, including emergency thoracic drainage, thoracentesis, and oxygen uptake, her symptoms improved. Considering pregnancy as an immune escape physiological process, the patient continued treatment with osimertinib, and a partial response (PR) lasting 16 months was observed. Therefore, this case highlights the importance of being vigilant about the rapid development of the tumor after delivery in pregnant patients with EGFR-mutation lung cancer and taking preventive measures to cope with various emergencies.

## Introduction

In recent decades, lung cancer has remained the most lethal type of cancer. According to the 2020 global cancer statistics (1), there were 1.8 million deaths from lung cancer, far surpassing other cancer types and posing a serious threat to people’s health and lives. While lung cancer frequently occurs in individuals over the age of 60, its incidence has been gradually increasing in the younger female population in recent years (2, 3). As women delay family formation due to social and economic development, tumors and pregnancy may occur simultaneously. Although the occurrence of malignant tumors during pregnancy is relatively rare, approximately 1 case per 1,000 pregnant women is diagnosed with malignancy (4). While lung cancer is one of the most common malignancies, it is rare during pregnancy. Non-small-cell lung cancer (NSCLC) is the most common histological type, accounting for 80%–85% of all lung cancers in pregnancy (5).

The epidermal growth factor receptor (EGFR) pathway is a crucial signaling pathway in NSCLC, and the discovery of EGFR mutations as the most significant oncogenic driver genes in NSCLC has revolutionized the treatment paradigm for patients with advanced NSCLC. The deletion of exon 19 and the L858R mutation in exon 21 are considered the “classical” EGFR mutations, accounting for approximately 85% of EGFR mutations in NSCLC (6). Several targeted EGFR tyrosine kinase inhibitors (TKI) have been developed, demonstrating promising efficacy in patients with EGFR mutations. Among them, the third generation TKI, osimertinib, has stood out (7).

The ethical dilemma of balancing the health of the mother and the fetus has garnered significant attention. If a woman decides to proceed with the pregnancy, treatment options can present a significant challenge (5, 8). A review of published literatures related to lung cancer during pregnancy has revealed more than 80 reported cases. The majority of patients had advanced or metastatic disease and experienced unfavorable outcomes. To our knowledge, few reports exist on the administration of third-generation EGFR inhibitors (osimertinib) to pregnant patients with lung cancer. The effects of osimertinib on the fetus and its efficacy in the treatment of lung cancer during pregnancy require further investigation.

Here, we present the first case report of a patient with metastatic non-small-cell lung cancer who experienced tumor progression as a result of an unplanned pregnancy during treatment with osimertinib. Following the patient’s artificial induction of labor, her condition rapidly deteriorated with malignant pleural effusion and dyspnea. However, she was able to continue treatment with osimertinib and experienced a sustained tumor response.

## Case presentation

**Figure 1:**
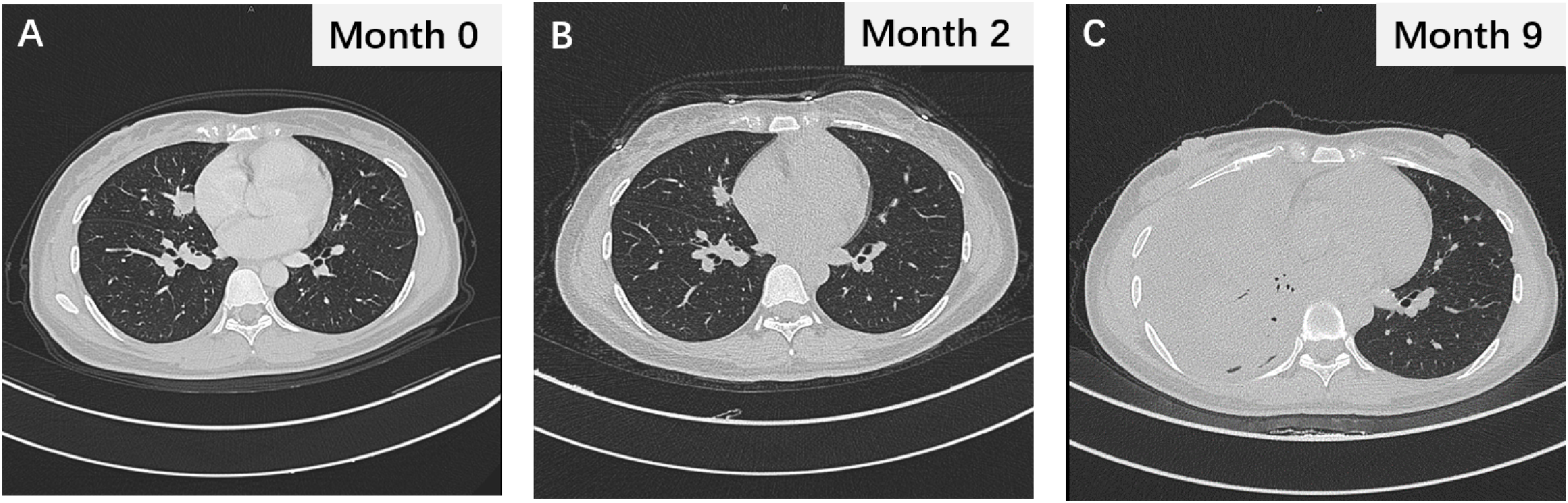
**(A)** The thoracic CT image displaying lung lesions during initial evaluation. **(B)** The thoracic CT scan showing the changes in lung lesions after 2 months of gefitinib treatment. **(C)** The chest CT image during gefitinib resistance.

A 29-year-old young woman who had never smoked presented with a history of chest pain and cough that lasted for 1 week. Physical examination revealed a body temperature of 36.9°C, a pulse rate of 105 beats/min, blood pressure of 103/69 mmHg, and respiratory rate of 22 breaths/min. Dual lung percussion was clear, and the breathing sound of both lungs was clear without any wetness. The serum tumor markers showed that the cancer antigen 15-3 (CA15-3) was 45.78 U/ml, and CA72-4 was 11.78 U/ml, while no abnormalities were observed in the remaining indicators. Chest computed tomography (CT) revealed a 2.5 × 2.2 cm solid nodule in the middle lobe of the right lung, along with multiple small nodules in both lungs, a small amount of pleural fluid on the right side, and several enlarged mediastinal lymph nodes (**Figure 1A**). The patient had menarche at the age of 14 with a menstrual cycle of 28 ± 3 days, lasting 3–5 days, normal menstrual volume and color, and no dysmenorrhea history. She was pregnant once, and a healthy male birth was born at the age of 27. The patient denied any history of previous tumor disease or family history of the same. Endoscopic ultrasound-guided fine-needle aspiration (FNA) pathology biopsy indicated scattered heterogeneous glandular epithelium. Immunohistochemistry (IHC) staining showed CK7 (+), CK20 (−), CDX2 (−), TTF-1 (8G7G3/1) (+), Napsin A (+), GATA3 (−), Ki67 (MIB-1) (+, 30%–40%), ALK-V (−), and ROS1 (−). These results supported the diagnosis of NSCLC of adenocarcinoma. Furthermore, second-generation genetic testing of the puncture specimen revealed a short deletion in exon 19 of the EGFR gene (del-19) and a mutation in L858R in exon 21. After a comprehensive evaluation, the oncologist assigned the staging as cT4N2M1a (IVa stage).

**Figure 2:**
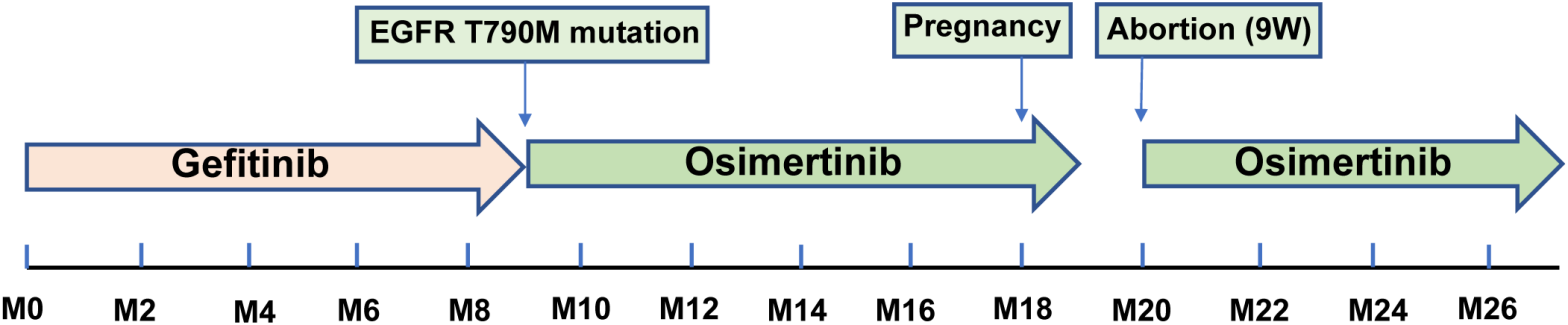
Flow-process diagram illustrating the patient’s tumor management.

Following the diagnosis of adenocarcinoma due to the EGFR mutation, the patient was prescribed the EGFR-TKI gefitinib at a dosage of 250 mg p.o. q.d (**Figure 2**). Two months after treatment, a thoracic CT scan indicated a significant reduction in the nodule in the middle lobe of the right lung, which had decreased to 1.5 × 1.0 cm, without enlargement of the hilar and mediastinal lymph nodes (**Figure 1B**). The efficacy assessment showed a partial response (PR), and gefitinib was continued. However, 9 months later, the patient again experienced chest pain and coughing up blood with dyspnea, serum carcinoembryonic antigen (CEA) was 91.22 ng/ml, and neuron-specific enolase (NSE) was 16.77 ng/ml, and CT imaging indicated partial pulmonary atelectasis and solid changes in the middle and lower lobes of the right lung, moderate pleural effusion on the right side, and an increase in bilateral lung lesions and regional lymph nodes (**Figure 1C**). The response evaluation revealed progressive disease (PD). Additionally, the follow-up genetic sequencing revealed the addition of EGFR T790M mutation in exon 20, which indicated the resistance to gefitinib.

**Figure 3:**
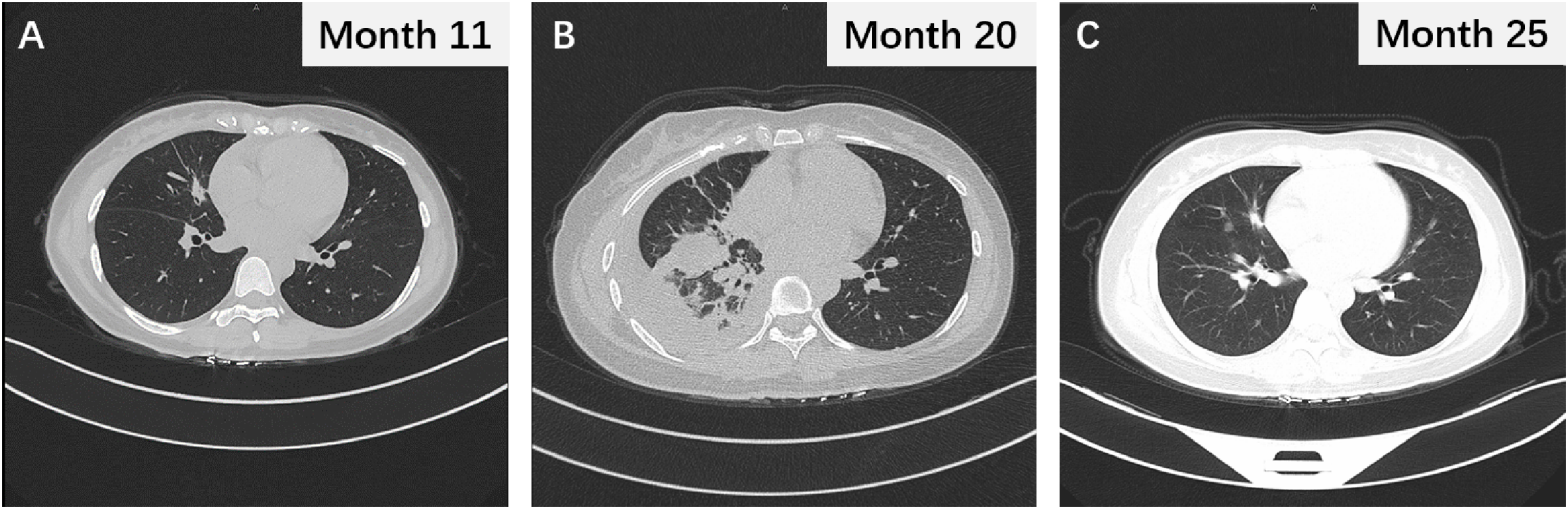
**(A)** The thoracic CT scan revealing the changes in lung nodules after 2 months of osimertinib treatment. **(B)** The chest CT image following termination of pregnancy. **(C)** Chest CT scan after 16 months of osimertinib treatment and demonstrating sustained partial response.

Following treatment with osimertinib, a third-generation EGFR-TKI, the patient’s target lesions exhibited significant reduction after 2 months of dosing. The bilateral lung nodules were observed to have shrunk and decreased, the right pleural effusion disappeared, and the right hilar and mediastinal lymph nodes shrank, as confirmed by **Figure 3A**. The efficacy of the treatment was assessed as PR. Continuous administration of osimertinib for 8 months further reduced the tumor. However, the patient was unexpectedly found to be 4 weeks pregnant, and pregnancy termination was recommended due to the risks associated with treatment using osimertinib. Despite being fully informed of the potential risks of preterm delivery, fetal malformation, and late developmental complications associated with osimertinib treatment, the patient and her family decided to continue with the pregnancy. Targeted therapy was suspended in order to avoid potential risks of osimertinib to healthy fetal development. Unfortunately, the disease progressed upon assessment at 8 weeks of pregnancy.

**Figure 4:**
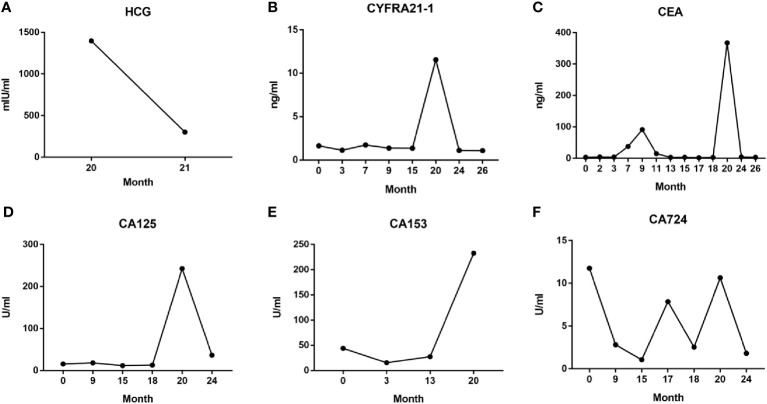
Serum HCG **(A)** and tumor markers CYFRA21-1 **(B)**, CEA **(C)**, CA125 **(D)**, CA153 **(E)**, and CA724 **(F)** levels during the entire diagnosis and treatment process.

We again informed the risks associated with continuing the pregnancy, and the patient ultimately decided to undergo an abortion at 9 weeks of gestation. However, an unforeseeable emergency arose after the procedure: the human chorionic gonadotrophin (HCG) levels rapidly dropped (**Figure 4A**). Shortly thereafter, the patient developed severe chest pain, respiratory distress, and moderate right pleural effusion. Red blood cell and lymphocyte counts decreased, and blood gas analysis revealed a pH of 7.452, oxygen level of 116.4 mmHg, potassium level of 3.37 mmol/L, glucose level of 6.60 mmol/L, oxygen saturation of 99.5%, and ion level of 1.02 mmol/L. Additionally, there was a significant increase in C-reactive protein (CRP), interleukin-6 (IL-6), and various tumor markers, indicating a rapidly deteriorating and urgent situation.

The patient was treated urgently with oxygen therapy and ultrasound-guided thoracentesis and drainage to relieve the pleural effusion. As gestation can lead to abnormal hormone secretion and immunosuppression, we believed that the rapid progression of the disease was not due to drug resistance to osimertinib. Therefore, 1 week after abortion, osimertinib targeted therapy was resumed, resulting in the disappearance of the pleural effusion and reduction in lung nodules and lymph nodes (**Figure 3B**). At the time of data collection, the patient had been on osimertinib treatment for over 16 months and was still benefiting from it (**Figure 3C**).

Throughout the course of treatment, we also monitored the patient’s serum tumor marker levels, the NSCLC-specific marker cytokeratin fragment 19 (CYFRA21-1) (**Figure 4B**), and the broad-spectrum markers CEA, CA125, CA153, and CA724 (**Figures 4C–F**). These data were concordant with imaging findings and patient symptoms, to some extent, allowing to monitor the therapeutic effect, assess prognosis, and predict recurrence (9–11). Especially in the 20th month, after abortion at 9 weeks of pregnancy, serum levels of the above five tumor markers increased sharply, reflecting the critical situation caused by rapid tumor progression at that time; when continued treatment with osimertinib, the patient’s serum tumor markers decreased rapidly, consistent with imaging findings (**Figure 3C**).

## Discussion

Lung cancer ranks third in prevalence among women and is the second most lethal tumor type worldwide (1). In recent years, lung cancer in pregnant women has been increasingly reported, and the majority of patients are diagnosed with progressive disease at stage III–IV (12). With advances in the study of lung cancer biology, molecular characteristics, and biomarkers, the treatment paradigm of lung cancer has undergone significant changes, leading to prolonged survival and improved the quality of life for patients (13). However, the management of pregnancy with lung cancer remains complicated. The challenge lies in balancing optimal treatment outcomes with fetal health, as treatment may potentially threaten the healthy development of the fetus, leading to serious ethical considerations (14).

We present a case report of a young non-smoking woman with NSCLC who had an unplanned pregnancy during osimertinib treatment, despite being advised to use strict contraception. After the diagnosis of tumor progression, the patient made the difficult decision to terminate the pregnancy. Unexpectedly, 2 weeks following the induced abortion, the patient developed severe chest pain and respiratory distress, and CT imaging revealed multiple patchy and solid shadows in the right lung, right pleural effusion, and enlarged mediastinal and bilateral axillary lymph nodes. Pulmonary vein embolism, pulmonary heart disease, and severe lung infection were all ruled out as potential causes, and the rapid progression of lung cancer was confirmed. However, with continued treatment of osimertinib, the patient achieved sustained remission.

In this case, three questions have arisen for our consideration. First is the efficacy and safety of drugs previously reported in patients with pregnancy-related driver gene-positive lung cancer, whether treated accordingly during pregnancy or after delivery, as compared to previous studies (15, 16). However, we noted that our report differed from previous cases, as most previous cases were diagnosed with lung cancer after pregnancy, whereas we reported an unplanned pregnancy during lung cancer treatment. After pregnancy, the body enters a state of immune escape from the fetus and placenta, which facilitates normal fetal growth and development. Unfortunately, tumor cells also simultaneously escape from immune cells with the help of the body’s state of immune suppression and grow rapidly (17). Additionally, the increased estrogen and progesterone levels during pregnancy, in response to estrogen receptor-positive tumor cells, promote the proliferation of lung cancer cells, which may contribute to rapid tumor progression (18).

Second, what is the cause of rapid disease progression in patients after undergoing induced abortion? After termination of pregnancy, the immune-evasion mechanism of the fetus and malignant tumor ceases, and the organism restores normal immune function. This recognition of mutated tumor cells induces a strong anti-tumor immune response in a short period of time, and a large number of immune cells infiltrate, leading to acute tumor growth and pleural effusion. Moreover, abortion is an exogenous physical trauma that causes an inflammatory response in the body, attracting inflammatory cells and secretion of a large number of inflammatory factors. The healing process of the trauma further increases the expression of epidermal growth factor (19), thus accelerating tumor deterioration. Additionally, it has been demonstrated in NSCLC cell lines that estrogen can induce downregulation of EGFR expression in tumor cells (20), and this finding was also verified in mouse experiments that the administration of exogenous estrogen to male mice significantly inhibited the growth of lung cancer and attenuated NF-κB-driven immunosuppression (21). The rapid decrease in estrogen and progesterone levels *in vivo* after termination of pregnancy and the liberation of NF-κB immunosuppression, along with the upregulation of EGFR expression in response to estrogenic changes, may also be important for the disease progression.

Third, does pregnancy have different effects on lung cancer with different driver genetic alterations? Our report is similar to two other cases of lung cancer in pregnant patients delivered by cesarean section, which exhibited rapid disease progression after delivery and followed a consistent clinical course (22, 23). One of the cases involved a 27-year-old woman who experienced fulminant respiratory failure on the third day after cesarean delivery. Despite the best supportive care, she succumbed to cardio-pulmonary failure 4 days postoperatively (23). Notably, all three lung cancer cases, including our own, exhibited amplification or mutation of the EGFR driver gene. Another driver oncogenic molecule, the anaplastic lymphoma kinase (ALK) gene, is more commonly implicated in pregnancy-associated lung cancers (8). However, unlike EGFR mutations, pregnancy-associated lung cancers carrying ALK gene alterations did not show acute tumor progression after delivery; sustained disease remission was achieved through continued ALK-TKI therapy (24, 25). The observed differences in tumor behavior before and after delivery in pregnancy-associated lung cancers with different driver gene alterations may be related to dominant oncogenic pathways. Patients with EGFR mutations may be more sensitive to changes in hormonal and inflammatory responses *in vivo*, indicating the need for individualized formulation of management measures for lung cancer in pregnancy with specific driver mutations.

Therefore, termination of pregnancy in lung cancer patients requires heightened vigilance, especially in cases associated with EGFR mutations, as the disease may worsen dramatically following cesarean section or abortion. Adequate preparations should be made before termination of pregnancy to handle any unexpected emergencies, such as oxygenation, blocking driver gene signaling, suppressing immune response, and reducing the inflammatory response. Currently, there are no established guidelines for the clinical management of lung cancer during pregnancy, and treatment decisions are mostly based on case studies. Therefore, we strongly advocate the establishment of an international mutual support network platform dedicated to the study of pregnancy-related lung cancer, which can provide references for the standardized treatment of pregnancy-related lung cancer by sharing valuable experiences and insights.

Furthermore, strict contraception during treatment for female lung cancer patients of childbearing age is of utmost importance, as pregnancy can severely limit treatment options and delay the optimal tumor treatment. For lung cancer diagnosed during pregnancy, the main conflict between disease control of the mother and potential risks to the fetus should be carefully considered when choosing an optimal treatment. To provide an individualized treatment strategy for mothers with lung cancer during pregnancy, a multidisciplinary and integrated collaborative approach consisting of oncologists, obstetricians, and psychologists should be established, meanwhile respecting the patient’s autonomous will.

## Patient’s perspective

Unexpectedly, I became pregnant while undergoing treatment for lung cancer. Despite being advised by my doctor about the possibility of treatment resistance, I chose to continue with the pregnancy. However, the disease progressed rapidly, and I made the decision to terminate the pregnancy. Following the procedure, I experienced a life-threatening complication that required prompt medical intervention. Thanks to the prompt and dedicated care provided by my medical team, the tumor was brought under control. I feel extremely fortunate to have received such excellent care.

## Data availability statement

The original contributions presented in the study are included in the article/supplementary material. Further inquiries can be directed to the corresponding author.

## Ethics statement

Written informed consent was obtained from the patient/guardian for all data and images in this study.

## Author contributions

YW conceptualized the idea of this case. QM performed data and drafted the manuscript. PS and KZ revised and edited the manuscript. All authors read and approved the final manuscript.
